# Pneumopathie interstitielle diffuse révélant la leucémie-lymphome à cellules T de l’adulte HTLV1+

**DOI:** 10.11604/pamj.2016.25.150.8619

**Published:** 2016-11-14

**Authors:** Nouama Bouanani, Mouna Lamchahab

**Affiliations:** 1 Service de Médecine, Centre Hospitalier Régional de Safi, Casablanca, Maroc; 2 Service d’Hématologie et d’Oncologie Pédiatrique, CHU 20 Août de Casablanca

**Keywords:** Leucémie/lymphome T de l´adulte, HTLV1, pneumopathie interstitielle, Adult T-Cell leukemia/lymphoma, HTLV1, interstitial lung disease

## Abstract

La leucémie/lymphome à cellules T de l'adulte est une prolifération tumorale de cellules lymphoïdes T matures activées, dont l'agent étiologique est le rétrovirus humain T cell-leukemia virus type 1, ce virus entraine rarement des désordres inflammatoires bronchioloalveolaires. Nous rapportons l'observation d'un patient hospitalisé pour une pneumopathie interstitielle diffuse et dont le bilan étiologique a révélé une leucémie lymphome à cellules T de l'adulte HTLV1+.

## Abstract (en)

Adult T-Cell Leukemia/Lymphoma is a tumoral proliferation of activated mature T lymphoid cells whose causative agent is a retrovirus known as Human T-cell leukemia virus type 1. This virus rarely causes inflammatory bronchioloalveolar disorders. We report the case of a patient hospitalized with diffuse interstitial lung disease and whose etiological assessment revealed adult T-Cell Leukemia/Lymphoma (HTLV-1).

## Introduction

L'HTLV1 (Human T cell-Leukemia Virus type 1) est l'agent étiologique de la leucémie lymphome à cellules T de l'adulte (ATL), ce virus entraine rarement des désordres inflammatoires bronchioloalveolaires notamment la pneumopathie interstitielle diffuse et la pan bronchiolite [1, 2]. Nous rapportons l'observation d'un patient hospitalisé pour une pneumopathie interstitielle diffuse et dont le bilan étiologique a révélé une leucémie lymphome à cellules T de l'adulte HTLV1+.

## Patient et observation

**Figure 1:**
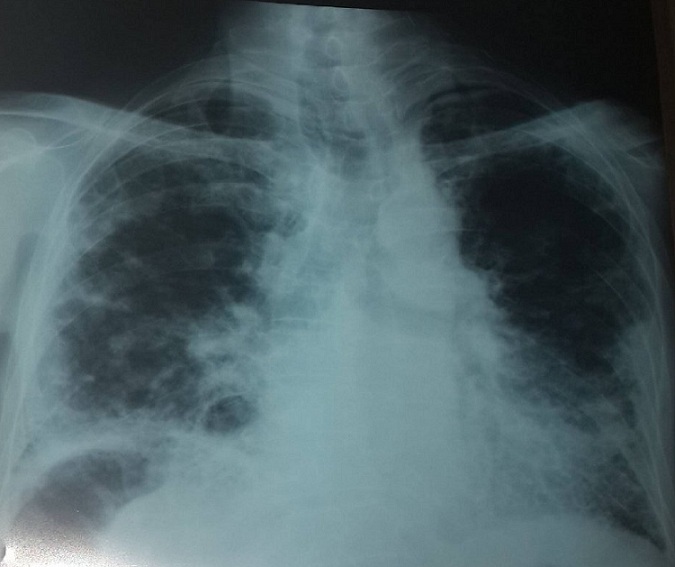
Radiographie thoracique face à l’admission: opacités réticulomicronodulaires diffuses à répartition périphérique et basale

**Figure 2:**
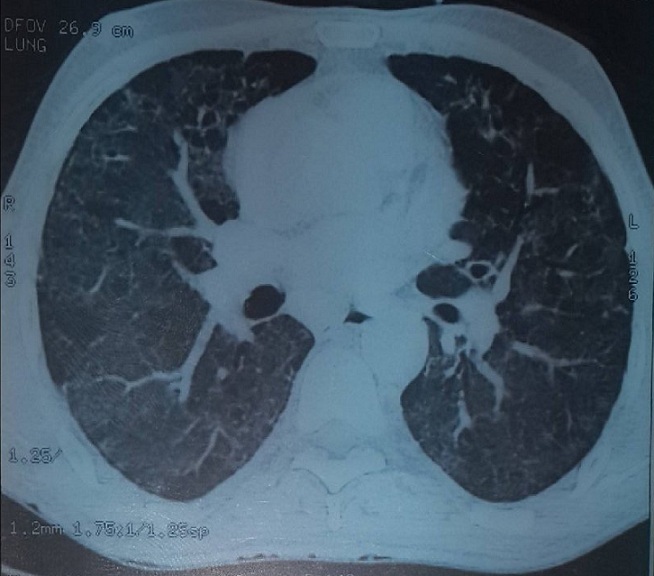
TDM thoracique: pneumopathie intersitielle diffuse avec aspect en verre dépoli

**Figure 3:**
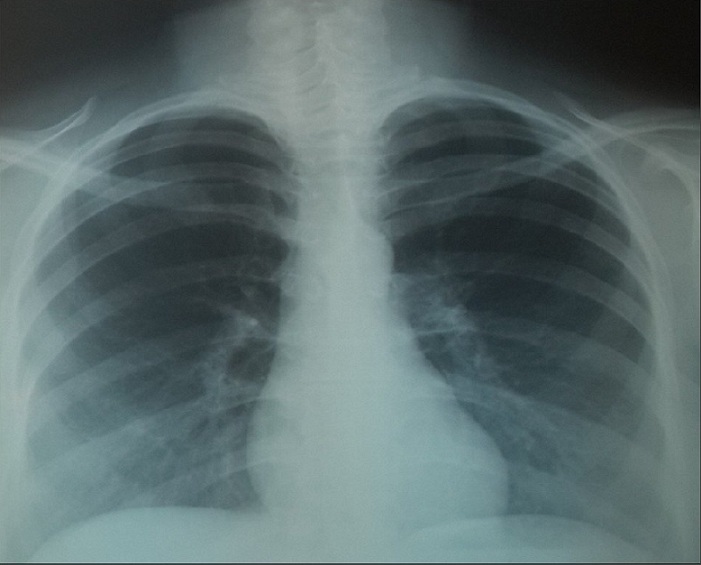
Radiographie thoracique sensiblement normale (après traitement)

Mr M.A âgé de 53 ans, Mauritanien, sans contage tuberculeux connu récent dans son entourage, sans habitudes toxiques et sans exposition professionnelle particulière. Le patient a présenté 3 mois avant son hospitalisation une toux sèche devenant productive sans hémoptysies associée à des douleurs thoraciques diffuses peu intenses et une dyspnée d'effort évoluant dans un contexte de sensations fébriles et d'altération de l'état général. Le patient a été admis au service de pneumologie, l'examen général a retrouvé une Performance Status à 1 sans hippocratisme digital avec à l'examen pleuropulmonaire des râles crépitants bilatéraux. Le reste de l'examen somatique a été sans particularité en dehors d'une adénopathie axillaire gauche de 1 cm/1 cm sans signes inflammatoires, mobile par rapport aux deux plans superficiel et profond. La radiographie du thorax (Figure 1) a objectivé de multiples opacités réticulo micronodulaires au niveau des deux champs pulmonaires prédominant au niveau des bases. La tomodensitométrie thoracique (Figure 2) a montré un aspect en verre dépoli diffus au niveau des deux champs pulmonaires faisant évoquer une pneumopathie interstitielle diffuse sans adénopathies médiastinales. Devant ce tableau nous avons discuté en premier lieu, sur un terrain d'immunodépression probable, une origine infectieuse notamment une tuberculose pulmonaire, une aspergillose semi invasive ou une infection à germes banals. Une détermination thoracique d'une maladie de système à savoir une sarcoïdose dans sa forme atypique ou une vascularite mais il n'y avait pas de signe d'appel clinique ou radiologique en faveur. En dernier lieu nous avons évoqué une détermination thoracique d'une hémopathie maligne. Vu le contexte endémique de tuberculose dans notre pays, l'urgence était de rechercher le Bacille de Koch (BK) dans les expectorations qui sont revenues négatives. La bronchoscopie souple a objectivé une inflammation de 2^ème^ degré diffuse de tout l'arbre bronchique. Le lavage broncho alvéolaire a montré un liquide inflammatoire à prédominance lymphocytaire (30%), la recherche de BK, de germes banals et d'aspergillus dans le liquide d'aspiration bronchique a été négative. Les biopsies bronchiques ont été en faveur d'un remaniement fibreux et inflammatoire modéré de la muqueuse sans signe de malignité ou d'inflammation spécifique. L'exploration fonctionnelle respiratoire a objectivé un trouble ventilatoire mixte. Sur le plan biologique l'hémogramme a montré une hyperleucocytose à 70000 éléments/ mm3 à prédominance lymphocytaire 53000 élément/mm^3^, le taux de polynucléaires neutrophiles à 13000 élément/mm^3^, le taux d'hémoglobine et de plaquette a été normale. La Cytométrie en flux sur le sang périphérique a été en faveur d'un syndrome lymphoprolifératif de type T avec positivité de CD2, CD3, CD4, TCR alpha beta, HLA DR et négativité de CD7, CD8 et CD25. Enfin, la biopsie de l'adénopathie axillaire a montré une localisation ganglionnaire d'un lymphome T. Devant ces éléments, un bilan complémentaire a été demandé: un myélogramme et une tomodensitométrie abdomino pelvienne qui sont revenus normales, le frottis sanguin n'a pas révélé de cellules de Sézary, le taux des lactico-déshydrogénase à 507 UI/L, les sérologies virales de l'hépatite B, C, cytomégalovirus et HIV ont été négatives. Le caryotype médullaire a été normal, la biopsie ostéo médullaire a montré une moelle de richesse normale sans cellules malignes. Devant l'origine subsaharienne du patient, la sérologie HTLV1 a été demandée et qui est revenu positive. Le diagnostic retenu a été une pneumopathie interstitielle diffuse dans le cadre d'une leucémie lymphome à cellules T de l'adulte HTLV1+. Le patient a été mis sous corticothérapie orale faite de prednisolone à la dose de 1 mg/kg/jour durant 1 mois avec une bonne amélioration clinique et radiologique (Figure 3), puis adressé au service d'hématologie pour entamer la chimiothérapie.

## Discussion

L'infection par le virus HTLV-1 (Human T cell-Leukemia Virus type 1) touche entre 15 et 25 millions de personnes dans le monde. Les zones de forte endémie sont le sud du Japon, l'Afrique intertropicale et le bassin Caraïbe [1, 3]. Sept génotypes viraux sont décrit, dont quatre principaux (A,B,C,D) [4, 5]. L'HTLV-1 se transmet assez difficilement dans les populations humaines et nécessite avant tout des contacts répétés. La transmission s'effectue de la mère à l'enfant, principalement par un allaitement prolongé de plus de six mois ou par voie sexuelle ou sanguine. La très grande majorité des sujets infectés par HTLV-1 reste asymptomatique. L'HTLV-1 est un rétrovirus oncogène responsable de la leucémie/lymphome à cellules T de l'adulte (ATL). La prévalence de l'ATL est de l'ordre de 2,5% chez les porteurs du virus, elle atteint les adultes après une longue période de latence, avec un pic d'incidence entre 50-69 ans, majoritairement les hommes, son pronostic reste sévère en raison de la résistance à la chimiothérapie et à l'immunosuppression profonde [6–8]. L'HTLV1 peut entrainer des désordres inflammatoires dans différents organes même en l'absence d'ATL [7]. Les désordres bronchioloalveolaires liées à l'HTLV1 (HABA) sont rares, ils regroupent la pan bronchiolite diffuse et la pneumopathie interstitielle non spécifique, ils sont dus à une réaction immunitaire à médiation pulmonaire entrainant localement une production d'interleukine à l'origine des atteintes pulmonaires qui peuvent être symptomatiques ou de découverte fortuite et qui se traduisent sur le plan radiologique par une image de miliaire micronodulaire avec un aspect en verre dépoli comme le montre notre observation, le lavage broncho alvéolaire montre un taux de lymphocytes élevé et la biopsie pulmonaire montre une infiltration lymphocytaire autour des bronchioles, ces désordres bronchioloalveolaires sont généralement corticosensibles [1, 3, 6].

## Conclusion

La Pneumopathie interstitielle secondaire à l'HTLV1 est parmi les étiologies rares des pneumopathies interstitielles diffuses, d'évolution favorable sous corticothérapie, la réalisation d'une sérologie HTLV-1 doit être systématique devant tout syndrome leucémique ou lymphomateux de type T chez les patients provenant d'une zone de forte endémie.
